# Editorial: Immunomodulatory Roles of Extracellular Vesicles in Autoimmune Diseases

**DOI:** 10.3389/fimmu.2022.725090

**Published:** 2022-03-18

**Authors:** Zhifeng Gu, Winston Patrick Kuo

**Affiliations:** ^1^ Research Center of Clinical Medicine, Affiliated Hospital of Nantong University, Nantong, China; ^2^ Infectious Diseases, Predicine, San Francisco, CA, United States

**Keywords:** extracellular vesicles, autoimmune diseases, diagnosis, immunomodulatory, pathogenesis, therapeutics

Autoimmune diseases reflect a breakdown in self-tolerance that results from defects in our complex immune system. The mechanisms are multifactorial and include a combination of cellular, genetic, epigenetic, and molecular components that result in phenotypic inflammatory responses in a variety of tissues and organs. Extracellular vesicles (EVs) have been shown to play a role in immunomodulation and pathogenesis of autoimmune diseases. This Research Topic provides a few reviews and manuscripts highlighting the immunomodulation and therapeutic potential of EVs in a variety of autoimmune diseases including rheumatoid arthritis (RA), Sjögren’s syndrome (SS), bullous pemphigoid (BP), type 1 diabetes mellitus (T1DM), systemic lupus erythematosus (SLE), inflammatory bowel disease (IBD), and antiphospholipid-associated diseases (APS).

## Immunomodulatory Effects of Mesenchymal Stem Cells and Mesenchymal Stem Cell-Derived Extracellular Vesicles in Rheumatoid Arthritis

RA is a chronic autoimmune disease affecting joints *via* pain, inflammation, loss of mobility, and eventually the erosion of joints, with no effective treatment to date. As reviewed in this Research Topic, extracellular vesicles derived from mesenchymal stem cells (MSCs) are more stable, less toxic, and more effective at transferring nucleic acids, proteins, and lipids from parent to recipient cells. MSC-EVs are multipotent progenitor cells with immunomodulatory properties that can be easily obtained and expanded rapidly, as well as a potential therapeutic strategy for RA (Liu et al.).

## Recent Advances in the Use of Exosomes in Sjögren’s syndrome

SS is a chronic multi-organ autoimmune disorder primarily effecting the exocrine glands. As reviewed in this Research Topic, ongoing extracellular vesicle studies in SS have been limited to tears and saliva, neglecting the investigation of novel biomarkers and potential therapeutic effects of EVs in SS from effected tissues and organs (Huang et al.).

## Assessment of the Characteristics and Associated Factors of Infectious Complications in Bullous Pemphigoid

BP is an autoimmune disorder of the skin characterized by blistering, urticarial lesions, and itching. In this retrospective study, Chen et al. demonstrated that inpatient BP patients are at risk of infectious complications, leading to comorbidities, due to higher doses of corticosteroids. They compared the risk factors of infection of inpatients and outpatients to develop preventative and treatment strategies.

## Commentary: Proinflammatory role of blister fluid-derived exosomes in bullous pemphigoid

Liu and Li proposed the potential use of EVs for the understanding the proinflammatory roles of BP for diagnostics. As shown in studies, concentrations of cytokines are elevated in BP, and it is speculated that EVs can transport the pathogenic autoantibodies associated with BP which can then be released and stimulate a favorable immune response.

## Molecular and Functional Diversity of Distinct Subpopulations of the Stressed Insulin-Secreting Cell’s Vesiculome

In this study, Giri et al. investigated the changes in the relative composition of the vesiculome as well as the partition of the candidate autoantigen insulin and immunostimulatory miRNA sequences inside apoptotic bodies, microvesicles, and exosome subpopulations derived from equal amounts of healthy and stressed beta cells and their impact on innate immune responses. They identified that beta small extracellular vesicles (sEVs) have been shown to drive innate and adaptive pro-diabetogenic immune responses, with a limitation in the molecular and functional diversity of EVs in the beta cell’s secretome, which necessitates further exploration.

## Extracellular Vesicles in Rheumatoid Arthritis and Systemic Lupus Erythematosus: Functions and Applications

In this review, Zhang et al. reviewed recent studies examining the roles of EVs in RA and SLE, both chronic autoimmune diseases but SLE affects multiple organs, in understanding their pathogenesis, diagnosis, and therapeutic potentials.

## Neutrophil Extracellular Traps Tied to Rheumatoid Arthritis: Points to Ponder

Neutrophils play a central role in our immune defense system with pathogen clearance, immune regulation, and disease pathology. Song et al. describe the role of neutrophil extracellular traps (NETs) in detail, as novel therapeutic targets for RA.

## Extracellular miR-574-5p Induces Osteoclast Differentiation *via* TLR 7/8 in Rheumatoid Arthritis

Hegewald et al. detail the roles of sEVs carrying microRNAs (miRs) in RA. The sEVs from synovial fluid promote osteoclast differentiation, attributed to high levels of extracellular miR-574-5p. They continue to demonstrate that enhanced osteoclast maturation is mediated by toll-like receptor (TLR) 7/8 signaling due to the mechanism of miR-574-5p binding. This is a novel finding of the role of miR-574-5p which may provide a therapeutic approach to protect osteoclast-mediated bone destruction in RA.

## Granulocytic Myeloid-Derived Suppressor Cell Exosomal Prostaglandin E2 Ameliorates Collagen-Induced Arthritis by Enhancing IL-10^+^ B Cells

In this comprehensive study, Wu et al. identified granulocytic-myeloid suppressor cell (G-MDSC)-derived EVs as a potential mediator in the treatment of mice with collagen-induced arthritis (CIA). The initial finding demonstrated lower arthritis index values and decreased inflammatory cell infiltration, indicating an alteration of the humoral environment by mediating high levels of prostaglandin E2 (PGE2), by production of IL-10^+^ B cells.

## Emerging Roles of Exosomes in T1DM

T1DM is caused by an immune-mediated destruction of pancreatic beta cells. Pang et al. detail a comprehensive review on the understanding of how exosomes can 1) enable the underlying pathogenic mechanisms of T1DM, 2) provide novel biomarkers for T1DM diagnosis, and 3) lead to the development of new TIDM therapeutic strategies.

## Olfactory Ecto-Mesenchymal Stem Cell-Derived Exosomes Ameliorate Experimental Colitis *via* Modulating Th1/Th17 and Treg Cell Responses

In this extensive study, Tian et al. have identified the immunoregulatory property of exosomes derived from olfactory ecto-mesenchymal stem cells (OE-MSCs) and their immunomodulation capacity to ameliorate disease severity in IBD mice, primarily by regulating Th-cell immune responses. Their study suggests OE-MSC exosomes are a potential novel cell-free therapy for targeting inflammatory diseases.

## Exosome-Contained APOH Associated With Antiphospholipid Syndrome

APS is a systemic autoimmune disorder in which the body’s immune system makes antibodies that attack phospholipids which can lead to thrombosis and/or pregnancy complications. Tan et al. conducted human and mouse studies to demonstrate that APS exosomes are a key factor in the pathogenesis of APS and that apolipoprotein H (APOH) is a protein that impairs vascular biological function. They concluded APS and APOH exosomes impair vascular development (pathogenesis) and lead to pregnancy complications, providing new targets for therapeutic intervention.

## Extracellular Vesicles Secreted by Mesenchymal Stromal Cells Exert Opposite Effects to Their Cells of Origin in Murine Sodium Dextran Sulfate-Induced Colitis

Tolomeo et al. compared the effects of MSCs and of MSC-EV administration in mice with colitis induced by dextran sulfate sodium (DSS). They reported naïve MSCs and induced MSC administration resulted in poor clinical and histological outcomes, with pro-inflammatory polarization of intestinal macrophages. However, when the mice were treated with induced EVs, there was decreased intestinal fibrosis and angiogenesis and a striking increase in intestinal expression of Mucin 5ac, suggesting improved epithelial function. EVs demonstrated a beneficial effect, more predictable behavior, a safer therapeutic profile, and efficacy with respect to their cells of origin.

## Conclusions

This Research Topic has provided examples in which EVs have displayed immunomodulatory roles in autoimmune diseases. In addition, it highlighted and suggested the necessity for future discovery of new targets for therapeutic interventions which will aid in the diagnosis, understanding the mechanisms, and outcomes for a variety of autoimmune diseases.

## Author Contributions

All authors listed have made a substantial, direct, and intellectual contribution to the work and approved it for publication.

## Conflict of Interest

WK is employed by Predicine.

The remaining author declares that the research was conducted in the absence of any commercial or financial relationships that could be construed as a potential conflict of interest.

## Publisher’s Note

All claims expressed in this article are solely those of the authors and do not necessarily represent those of their affiliated organizations, or those of the publisher, the editors and the reviewers. Any product that may be evaluated in this article, or claim that may be made by its manufacturer, is not guaranteed or endorsed by the publisher.

